# Editorial: Plant ecophysiology: responses to climate changes and stress conditions

**DOI:** 10.3389/fpls.2025.1677128

**Published:** 2025-09-11

**Authors:** Peter Petrík, Yanbo Hu, Georgios Koubouris, Raul Antonio Sperotto, Srdjan Stojnic

**Affiliations:** ^1^ Chair of Forest Botany, Institute of Forest Botany and Forest Zoology, Technical University of Dresden (TUD), Tharandt, Germany; ^2^ College of Life Science, Northeast Forestry University, Harbin, China; ^3^ Hellenic Agricultural Organization (ELGO) DIMITRA, Institute of Olive Tree, Subtropical Crops and Viticulture, Chania, Greece; ^4^ Biology Institute, Botany Department, Graduate Program in Plant Physiology, Federal University of Pelotas, Pelotas, Brazil; ^5^ Institute of Lowland Forestry and Environment, University of Novi Sad, Novi Sad, Serbia

**Keywords:** abiotic stress, biotic interactions, drought stress, heat stress, stress resilience

## Introduction

**Figure 1:**
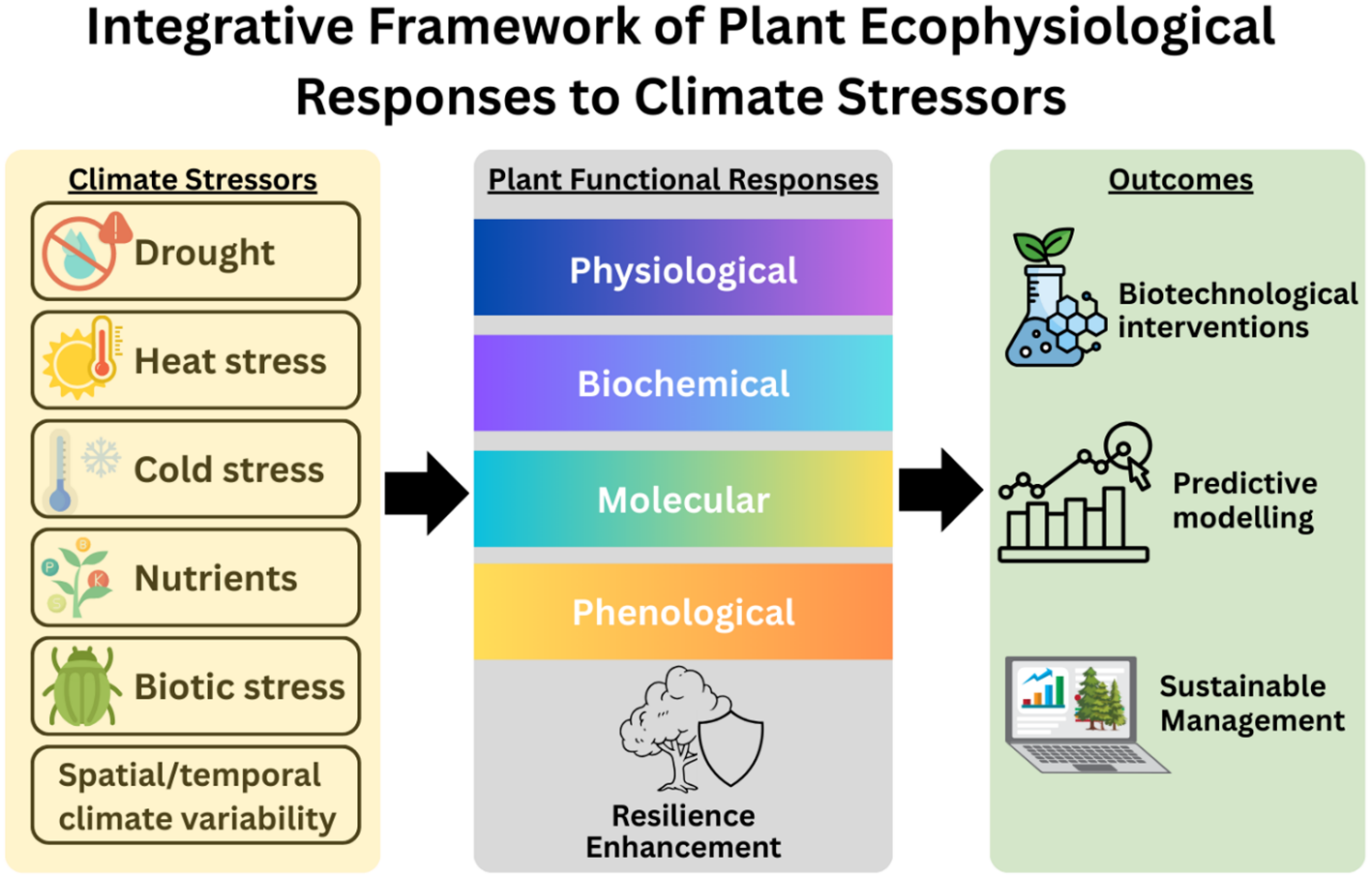
Conceptual framework illustrating the interconnected pathway between major climate-related stressors (left), plant ecophysiological responses (centre), and applied outcomes (right). This synthesis captures key mechanisms and themes presented in the Research Topic, including drought, temperature extremes, nutrient dynamics, biotic stress, and climate variability. Arrows denote the flow of influence, highlighting how physiological, biochemical, and molecular responses underpin advances in forecasting, biotechnological innovations, and resilience-oriented ecosystem management under climate change.

In an era of accelerating global climate change, understanding the physiological responses of plants to diverse environmental stresses is pivotal for predicting ecosystem resilience and ensuring sustainable agriculture and forestry practices (Dutta et al., 2020). Climate-driven alterations such as drought, flooding, temperature extremes, and nutrient fluctuations challenge plant survival and productivity, thereby threatening global biodiversity, food security, and ecosystem stability (Altman et al., 2024; Yuan et al., 2024). Given these pressing concerns the study of plant responses and adaptations to environmental conditions within plant ecophysiology framework, has become indispensable. This Research Topic brings together an array of studies that provide novel insights into plant performance under multiple abiotic and biotic stress scenarios. The compiled works illuminate how plants perceive, respond to, and recover from environmental stressors through intricate physiological, biochemical, and molecular mechanisms. Spanning a diverse range of species, ecosystems, and methodological approaches, these contributions collectively enhance our understanding of plant stress biology and inform practices aimed at mitigating climate change impacts. The papers included in this Research Topic are structured around different critical themes reflecting contemporary research priorities. Specifically, we present studies focusing on drought and waterlogging stress, temperature extremes, biotic stress interactions, nutrient dynamics, biotechnological strategies for stress mitigation, spatial and temporal climatic effects, and predictive analyses concerning climate change impacts on species distribution (**Figure 1**). This integrative approach not only provides a comprehensive snapshot of current research trends but also fosters interdisciplinary collaboration crucial for addressing complex ecological challenges.

## Drought and waterlogging stress

Drought and waterlogging represent significant abiotic stressors severely impacting plant health, productivity, and survival. Plants adapt to these conditions through diverse physiological and biochemical responses, as exemplified in this Research Topic by several studies. Zhang et al. explored growth and non-structural carbohydrate response patterns in *Eucommia ulmoides* under combined salt and drought stress, providing insights into carbohydrate metabolism during dual stress conditions. Cheng et al. used a process-based model to investigate how altered precipitation patterns influence grassland aboveground net primary productivity and precipitation utilization efficiency, underscoring differential ecosystem responses to climatic variability. Zhang et al. evaluated growth, physiological responses, and drought resistance in various flue-cured tobacco varieties, highlighting varietal differences critical for crop improvement under drought conditions. Ge et al. demonstrated how drought stress induces contrasting changes in organ carbon and soil organic carbon, enhancing resistance mechanisms in moso bamboo. Alipour et al. integrated metabolomic, transcriptomic, and phytohormonal analyses in *Thymus* species under water stress and foliar abscisic acid application, revealing complex molecular interactions involved in drought stress adaptation. Huang et al. investigated the effects of irrigation frequency on root growth, nutrient accumulation, yield, and water use efficiency of *Panax notoginseng* under micro-sprinkler irrigation, offering valuable strategies for sustainable cultivation practices. Knüver et al. showed that stress dose significantly explains drought recovery patterns in Norway spruce, contributing to understanding tree resilience in forest ecosystems. Finally, Calabritto et al. conducted physiological and image-based phenotyping to assess waterlogging responses in three kiwifruit rootstocks and grafting combinations, identifying rootstock-specific tolerance mechanisms crucial for orchard management under flooding conditions. Collectively, these studies provide comprehensive insights into plant responses and resilience strategies under drought and waterlogging stress, vital for developing adaptive management practices under changing climatic conditions.

## Extreme temperature stress

Temperature extremes, encompassing both cold and heat stress, present critical challenges to plant growth, reproduction, and survival. Plants respond to temperature fluctuations through complex regulatory pathways that involve physiological, biochemical, and molecular adaptations, all aimed at maintaining homeostasis under adverse conditions. Zhu et al. reviewed the mechanisms by which plants perceive temperature changes, focusing on thermosensors that mediate stress adaptation responses. This comprehensive exploration highlighted the molecular processes enabling plants to sense and respond effectively to temperature variations, thus underscoring the potential for developing crops resilient to climate change-induced temperature fluctuations. Padhiar et al. investigated the differential resilience of chickpea’s reproductive organs to cold stress across developmental stages. By elucidating antioxidant strategies employed to mitigate cold-induced damage, this study offered insights crucial for breeding programs aimed at enhancing fertility under cold stress conditions. Furthermore, Dong et al. demonstrated that exogenous application of 24-epibrassinolide significantly mitigates damage in grape seedlings exposed to low-temperature stress. The findings emphasize the potential of brassinosteroids in promoting plant tolerance to chilling stress, providing practical implications for improving grapevine cultivation in cooler climates. Collectively, these studies illuminate crucial mechanisms underlying plant responses to temperature stress, revealing opportunities to enhance crop tolerance and productivity amid increasingly erratic and extreme temperature regimes associated with climate change.

## Biotic stress

Biotic stress, involving pathogen infections, pest attacks, and interactions with various organisms, significantly influences plant growth, health, and yield. The ability of plants to effectively respond to these challenges through physiological and molecular adjustments is fundamental to maintaining productivity and sustainability in agriculture and forestry. Negesa et al. evaluated lowland coffee genotypes for resistance against leaf rust and wilt diseases in southwestern Ethiopia. They identified genotypic differences in disease susceptibility, thereby providing essential information for breeding programs targeting improved disease resistance and enhanced yield stability in coffee cultivation. Shafi et al. employed an integrative approach combining ecophysiological assessments with omics technologies to unravel the complex responses of crops to combined drought and herbivory stress. This holistic methodology revealed novel insights into how plants simultaneously manage abiotic and biotic stressors, offering potential pathways for developing robust, stress-resilient cultivars. These contributions underscore the importance of understanding plant responses to biotic stressors in the broader context of climate-induced challenges, ultimately guiding the development of integrated management strategies that enhance crop resilience and sustainability in agricultural systems.

## Nutrient dynamics under stress and climate change

Nutrient availability is a critical determinant of plant growth, productivity, and ecosystem function, significantly influencing how plants respond to environmental stresses. Several studies in this Research Topic provide insights into plant responses to nutrient dynamics under varying climatic and environmental conditions. Cai et al. explored nitrate nitrogen uptake and metabolism in the invasive species *Mikania micrantha*, shedding light on the physiological mechanisms underpinning its rapid growth and invasiveness. This research underscores the complex relationship between nutrient metabolism and ecological competitiveness in invasive plant species. Chen et al. investigated how elevation and seasonality modulate leaf decomposition rates and nutrient fluxes in karst river systems across China. Their findings highlight the significant interplay between environmental gradients, species diversity, and nutrient cycling, offering critical insights into ecosystem function and biodiversity conservation strategies. Li et al. reported distinct responses of canopy and shrub leaves to nitrogen and water addition in warm temperate forests, revealing intricate nutrient-use strategies and functional adaptations of plants under altered resource availability. Du et al. conducted a global analysis of plant nutrient limitation in response to atmospheric nitrogen and phosphorus deposition. This comprehensive study emphasizes the far-reaching implications of anthropogenic nutrient deposition on plant growth constraints and ecosystem stability on a global scale. Zhao et al. examined adaptive growth strategies of *Quercus dentata* under combined drought stress and nitrogen enrichment, elucidating physiological and biochemical mechanisms that confer resilience under multiple stressors, relevant for forest management and restoration efforts. Finally, Yang et al. assessed how soil and community factors influence the yield and medicinal quality of *Artemisia argyi* at varying altitudes of Funiu Mountain. The results provide essential knowledge for optimizing cultivation practices that enhance both yield and quality in medicinal plants grown under different environmental conditions. Collectively, these studies underscore the fundamental role of nutrient dynamics in shaping plant ecological strategies and stress responses, offering vital information for ecosystem management and agricultural productivity under changing climatic conditions.

## Biotechnological stress mitigation

Biotechnological approaches offer innovative solutions for enhancing plant resilience to environmental stresses, holding significant potential for sustainable agriculture and forestry practices. In this Research Topic, several studies demonstrate cutting-edge strategies leveraging biotechnology to alleviate plant stress responses and improve productivity. Kumar et al. investigated a methane-derived microbial biostimulant, demonstrating its efficacy in reducing greenhouse gas emissions while simultaneously enhancing rice yield. This novel biotechnological approach provides a promising avenue for sustainable rice cultivation that aligns agricultural productivity with climate change mitigation objectives. Ye et al. explored the interactions between root endophytic microorganisms and the negative ion release capacity of *Phalaenopsis aphrodite* under high-temperature stress. Their findings revealed the potential for manipulating root microbiomes to enhance heat stress tolerance in ornamental plants, offering practical implications for horticulture under warming climates. Wang et al. demonstrated the beneficial effects of exogenous application of 24-epibrassinolide in enhancing wheat resistance to dry-hot wind stress during grain filling. Their research highlighted improvements in hormone balance and flag leaf photosynthetic performance, suggesting brassinosteroids as viable candidates for managing climate-related abiotic stress in cereal crops. Finally, Deng et al. elucidated the role of the *skp1* gene of the SCF complex in lipid metabolism and abiotic stress responses in the model organism *Chlamydomonas reinhardtii*. By providing insights into fundamental molecular pathways involved in stress adaptation, this study paves the way for targeted genetic manipulation aimed at enhancing stress tolerance in microalgae and potentially plant species. These studies collectively underscore the transformative potential of biotechnological interventions for mitigating plant stress, offering practical tools and strategies to bolster plant resilience and productivity in an era of increasing environmental unpredictability.

## Spatial and temporal climate effects

Understanding how spatial and temporal climatic variability influences plant physiological responses is critical for predicting ecosystem dynamics under changing climate scenarios. Studies in this Research Topic contribute valuable insights into how plants adapt their functional traits across different climatic gradients and seasons. Sanchez et al. examined seasonal ecophysiological responses of two páramo species, highlighting that light availability predominantly shapes plant functioning rather than water limitations. This research underscores the significance of seasonal environmental fluctuations in shaping physiological adaptations in high-altitude ecosystems. El-Barougy et al. investigated factors shaping beta diversity in arid landscapes, focusing on the synergy of climate, soil properties, and species traits. Their findings emphasize the importance of understanding climatic and edaphic influences on biodiversity patterns, essential for effective management and conservation strategies in arid regions. Robin et al. explored interactions between leaf phenological types and functional traits in determining variations in isoprene emissions from central Amazon Forest trees. This study provides essential data on how plant functional diversity affects ecosystem-level responses to climatic factors, particularly relevant in the context of atmospheric chemistry and climate feedback mechanisms. Blinkova et al. analyzed the impacts of limiting environmental conditions on the functional traits of vegetative shoots in *Hedera helix*. Their research sheds light on adaptive trait plasticity under environmental stress, relevant for understanding species resilience and informing urban greening and ecological restoration practices. Collectively, these studies illuminate how spatial and temporal climatic variations profoundly influence plant ecophysiology, informing strategies for ecological forecasting and adaptive ecosystem management amid climate change.

## Ecophysiological forecasting in changing climate

Climate change profoundly influences plant distributions, physiology, and overall ecosystem health, driving shifts in species suitability and productivity. Predictive modelling and physiological assessments presented in this Research Topic enhance our understanding of plant responses under future climate scenarios, guiding adaptation and mitigation strategies. Xu et al. employed the MaxEnt model to project changes in habitat suitability for *Hibiscus syriacus* in China, providing critical insights into potential shifts driven by future climate conditions. Similarly, Wu et al. predicted suitable geographical areas for *Pulsatilla chinensis* under current and anticipated climatic scenarios, highlighting species-specific responses to climate variability. Guillamon et al. unraveled metabolic alterations in peach trees under agrochemical treatments during flower bud endodormancy in response to global warming, revealing biochemical mechanisms underlying adaptation to changing winter conditions. Singh et al. provided morphological and pomological characterizations of bael (*Aegle marmelos*) genotypes to identify cultivars with enhanced resilience suitable for climate change mitigation in the north-western Himalayas. Wen et al. utilized MaxEnt modelling to forecast the potential distribution of the medicinal plant Astragali Radix in China, offering predictive insights crucial for conserving medicinal plant resources under climate change. Lu et al. similarly assessed climate change impacts on *Clinopodium polycephalum* distribution, integrating temporal data, ArcGIS, and MaxEnt models to project future distribution patterns and conservation priorities. Shang et al. identified key traits influencing resistance to wind damage in *Eucalyptus camaldulensis* in coastal areas of South China, providing valuable data for forestry management under increasingly severe weather events. Lastly, Zhang et al. analyzed distribution patterns and dynamic niches of *Magnolia grandiflora* in both USA and China under climate change scenarios, facilitating strategic planning for urban greening and biodiversity conservation. Collectively, these studies underscore the critical need to integrate predictive modelling with physiological and biochemical analyses to anticipate plant responses and inform adaptive management practices in an era of accelerating climate change.

## Concluding remarks and future perspectives

This Research Topic provides comprehensive insights into plant ecophysiological responses across diverse stress conditions driven by climate change, illustrating critical physiological mechanisms, adaptive strategies, and predictive frameworks. The breadth of research presented underscores the complexity of plant-environment interactions and highlights significant advancements in our understanding of plant resilience, stress adaptation, and ecosystem stability. Collectively, these studies reinforce the importance of integrating multidisciplinary approaches, combining physiological analyses, molecular techniques, ecological modelling, and biotechnology to enhance plant performance under current and future climate scenarios. However, significant gaps and challenges remain, including understanding complex interactions among multiple stressors, predicting long-term ecosystem responses, and developing practical strategies for sustainable agriculture, forestry, and biodiversity conservation. Future research should focus on bridging laboratory findings with field conditions, validating predictive models, and exploring genetic, epigenetic, and microbial avenues for stress mitigation. Additionally, an emphasis on collaborative, interdisciplinary approaches is essential to effectively address complex ecological challenges posed by accelerating global change. In conclusion, continued advancement in plant ecophysiology research remains crucial for fostering resilient ecosystems, ensuring agricultural sustainability, and mitigating the multifaceted impacts of climate change. The collective knowledge presented here not only informs current management practices but also lays the foundation for innovative strategies to sustainably manage plant resources in a rapidly evolving global environment.
